# Mapping the Language of Male Partner Violence: An Historical Examination of Power, Meaning, and Ambivalence

**DOI:** 10.1177/10778012231192571

**Published:** 2023-08-07

**Authors:** Christina Vogels

**Affiliations:** School of Communication Studies, AUT University, Auckland, New Zealand

**Keywords:** male partner violence, language, discourse, Bourdieu, destabilization

## Abstract

This article questions why violence carried out by men toward their female romantic partners remains so prevalent today by examining how it has been understood and talked about over time. The aim here is twofold: the first is to historically map the changes in how male partner violence has been addressed in society—and to what effect. The second is to examine subtle dynamics within this historical map in order to suggest how language could be used to destabilize its fixture in society.

## Introduction

Male partner violence—defined in this article as violence that is carried out by men toward their female romantic partners—has long been a fixture in society, with historians noting its presence at least as far back as the Roman Era (Sewell, 1989). Even with this permanent fixture, society still does not have a well-established cogent language to fully address women's experiences of this gendered form of violence (Easteal et al., 2012; Marcus, 1994; Minow, 1990). This article interrogates why this form of violence is so ubiquitous by historically mapping discursive patterns of how it has been understood and talked about in society. This article will chart this complex terrain of language (Wetherell & Potter, 1992) with two key aims: the first is to make more sense of why male partner violence is still so prevalent today and the second is to examine subtle dynamics within this historical map in order to suggest how language could be used to destabilize its fixture in society. To do this work, a Bourdieusian approach will be used to interrogate the effects that language has on how society makes sense of male partner violence and in turn, how this has impacted the support women experiencing this form of violence have been able to access. This approach will also be used to look at how ambivalence signals ways in which male partner violence could be destabilized.

## Violent Male Partners

The ways in which male partner violence has been understood and talked about have endured a complex history, layered in contradictory and confusing discourses (e.g., Amussen, 1994; Dobash & Dobash, 1992; Faulk, 1974; Gordon, 1988; Lentz, 1999; Lion, 1977; Moore, 2002; Peterson del Mar, 1996; Pleck, 1983; Schechter, 1982; Sewell, 1989; Snell et al., 1964). More often than not, misogynistic and gender-neutralizing understandings of this form of violence have dominated language. While feminists, through-out the ages, have played a key role in destabilizing these understandings, society is still no closer to having cogent language to discuss the problem. For instance, popular terminologies like “domestic violence” and “family violence” present a gender-neutral and ambiguous discourse regarding the gendered nature of male partner violence (Easteal et al., 2012; Marcus, 1994; Minow, 1990), which arguably impacts women's lived experiences of this form of violence and their ability to seek support.

Language used to discuss this problem, therefore, needs to transgress these dominant discourses. As Alcoff and Gray (1993) outline, language becomes transgressive when it exposes something that is “antithetical to the dominant discourse” (p. 268). They use the example of marital rape to illustrate their point:

Given that such terms as the “husband” have historically been defined as the man to whom a woman has given unconditional sexual access, the term “husband rapist” will necessarily transform our previous understandings of the terms “husband” and “rapist,” which in turn will affect how we understand “wife” “woman,” “sexuality,” “heterosexuality,” and even “man.” The formation rules that determine the generation of statements and that tell speakers how and in what circumstances they can meaningfully form and utter specific statements about sexual violence will also be affected.

Being able to utter the words “husband rapist” disrupts dominant discourses that give men “unconditional sexual access” to their wives by making husbands raping their wives a discursive reality. In turn, this brings the gendered nature of marital rape unambiguously to the forefront. For the purposes of this article, mobilizing the term *violent male partner* does similar discursive work. Being able to identify the doer—the violent male partner—not only holds men accountable, but it also disrupts nongendered understandings that proliferate within labels like domestic and family violence. Identifying the violent male partner also opens up discursive space to see the problem as not only confined to domestic setting—meaning that women who do not live with their romantic partners can become discursively included in language of this kind.

The specific use of the word “violent” is also discursively important here. One thing that can hinder language's cogency is the presence of inconsistent terminology. With regard to male partner violence, the words “abuse” and “violence” have often been used interchangeably, which has contributed to further ambiguity about what the problem actually is. Arguably, the word “violence” is more discursively useful than the word “abuse”: “abuse” is a broad, catch-all term used to define any “misuse” of something, whereas the word “violence” is more direct and captures the specific doing of violence toward another person, which is popularly defined as “inflict[ing] harm or injury upon” another (Oxford English Dictionary, n.d).

The word “harm” is particularly useful when defining the many facets of male partner violence. “Harm,” by definition, allows researchers to have a word that offers a complex array of understandings around male partner violence. In its verb formation, “harm” means “to damage or injure physically or mentally” (Merriam-Webster, n.d). This allows space to be opened up to see male partner violence as including harm inflicted via physical (be it sexual and/or nonsexual physical harm) as well as nonphysical acts of violence—such as using intimidation, gas-lighting, controlling another's physical movements, and controlling another's access to financial resources. And, importantly having the word “harm” at the center of defining the violence inflicted by male partners discursively recognizes the damage this form of violence does to women.

Therefore, by discursively locating the doer of this form of gendered violence as a *violent male partner,* the problem itself can then be termed *male partner violence* (see also Towns & Adams, 2000 for how this term is used by other scholars). *Male partner violence* is a more accurate term to use than say “domestic violence” or “family violence” as it provides both a gendered and unambiguous framework for talking about the problem—which is any form of violence used by a man to inflict harm on his (hetero)romantic partner.

## Authorized Language

This article critically examines the patterns of language used to talk about male partner violence to suggest ways in which misogynist and nongendered discourses could be disrupted today. While analyses of this sort are commonly undertaken by “discourse analysis” methodologies (e.g., Fairclough, 2013; Foucault, 1977), a Bourdieusian approach is being adopted in this article due to its unique way of accounting for how language develops over time within a complex interplay of structural power (fields) and subjectivity (habitus).

Pierre Bourdieu's (1930–2002) conceptual contributions are renowned for providing an innovative bridge between objectivist and subjectivist theory, which he achieved principally by introducing the concepts of habitus and field within a theory of practice to social theory (see Bourdieu, 1977, 1990; Bourdieu & Wacquant, 1992). As Adkins (2004, p. 193) elaborates, “Bourdieu understands the social world to comprise of differentiating but overlapping fields of action” that he often likens to “games” (e.g., see Bourdieu & Wacquant, 1992, p. 98), which are “patterned system[s] of objective forces…which impose on all the objects and agents who enter it” (Bourdieu & Wacquant, 1992, p. 17). The subjective realm of Bourdieu's understanding of the social is referred to as habitus. Habitus points to the internalization of objective structures (fields) that enter “into people's bodies and become[s] their identity” (Joas & Knob, 2011, p. 24). Bourdieu (1984, p. 466) maintains that habitus “instils a sense of place” within individuals, working as “a system of durable, transposable dispositions” (Bourdieu, 1990, p. 53) that exist in “dynamic relations” to fields (Messner, 2000, p. 459). With these understandings of how society is ordered (fields) and lived subjectively by individual agents (through habitus), Bourdieu adds a theory of practice to his framework in order to explain how individuals act in the world around them.

First and foremost, Bourdieu's position is clear: habitus is always “constituted in moments of practice” (Webb et al., 2002, p. 38). For Bourdieu, practice has many forms including “competencies, know-how, dispositions, perceptions” (Adkins, 2004, p. 194). In keeping with his theorizing of habitus, Bourdieu's understanding is that practices “operate below the level of consciousness and language through a ‘feel for the game’” (Adkins, 2004, p. 194). Bourdieu's treatment of practice, Adkins (2004) explains, is complex and layered, signaling his interest in the unconscious and prereflexive elements of practice, which involves “the ability to function effectively within a given field, an ability which cannot necessarily be articulated as conscious knowledge: knowing how rather than knowing that” (Lovell, 2000, p. 12). Here, Bourdieu provides us with a way to understand how language *takes form:* first, individual agents make meaning as they move throughout different fields in their lives via a prereflective taking up of “competencies, know-how, dispositions, perceptions” (Adkins, 2004, p. 194). These takings up of “competencies, know-how, dispositions, perceptions,” then, inform or constitute how agents talk and make meaning about and articulate the world around them.

As such, Bourdieu (1977, p. 21, *my emphasis*) argues that:

The constitutive power which is granted to ordinary language lies not in the language itself but in the group which authorizes it and *invests it with authority*. Official language particularly the system of concepts by means of which the members of a given group provide themselves with a representation of their social relations, sanctions and imposes what it states, tacitly laying down the dividing line between the *thinkable and unthinkable*, thereby contributions towards the maintenance of the symbolic order from which it draws authority.

This article will look at how the language surrounding male partner violence has taken form over the years in order to assess *who* has authorized its meaning and “invest[ed] it with authority” (Bourdieu, 1977, p. 21) at various periods in history. Following a Bourdieusian approach, I will look at which dominant fields at the time structured these authorized voices, and importantly how these either silenced or gave voice to women experiencing this form of violence. Here, it is important to ascertain when in history male partner violence has been “unthinkable” resulting in the problem being ignored, minimized, or silenced versus when it has become “thinkable” and, therefore, publicly recognized as having harmful consequences on women. Both of these *discursive* states (thinkability and unthinkability) impact one's subjectivity—or habitus (Bourdieu & Wacquant, 1992).

This intersection between *how* male partner violence is authorized by dominant groups and the subjectivity of women who are experiencing this form of violence *will* have consequences depending on which dominant group is vocal at the time. As will be discussed in more detail shortly, when feminist groups have taken control of the meaning of male partner violence and brought a *critical gendered discourse* to understanding the problem to a public platform, women have been *more* able to access appropriate help and support; when other dominant groups set on privileging patriarchal power have authorized the discourses around male partner violence, women have faced more barriers and even persecution around finding appropriate help and support. These differences in who owns the discourse around male partner violence have also impacted the consequences (or lack of) that violent male partners have faced at certain times. Mapping these differences in how male partner violence has been authorized—and therefore given meaning—in society matters (Amussen, 1994; Gordon, 1988; Hunt, 1992). Such a historical overview tells us how the problem has been addressed at different times in history, and importantly which authorized voices have controlled the agenda of how male partner violence has been spoken about.

Importantly, for contemporary researchers like myself working in this space, this map also provides contextual information about how male partner violence has been understood in the past, in order to look critically at how the problem is being dealt with today (see Pagelow, 1981). Here, specifically looking for patterns in how language has been in flux, provide insights into how male partner violence could be destabilized. To do this work, Lisa Adkins (2004) feminist adaptation of Bourdieu's theorizing of social change is particularly useful. Like a number of feminist scholars (see McLeod, 2005; McNay, 1999), Adkins’ (2004, p. 192) theory of what she terms “gender detraditionalization” starts with an overarching critique of Bourdieu's inconsistent account of social change. Principle to Bourdieu's overarching social theory is that the habitus submits to the field which enables the incorporation of social norms and thus mimesis to “work” (Adkins, 2004, p. 207). When speaking of change however, Bourdieu then breaks away from this understanding of mimesis—and in doing so, his theory of practice, as prereflexive and precognitive—to account for moments when social norms can be challenged by individuals within the social world (Adkins, 2004).

Adkin's (2004) framework for explaining gender detraditionalization starts with redefining reflexivity as not necessarily the “awakening of consciousness” that Bourdieu proclaims (see Bourdieu & Wacquant, 1992, p. 133). Instead, she (2004, p. 204) suggests that “reflexive practices may be said to be so habituated that they are part of the very norms, rules and expectations that govern gender in late modernity, even as they may ostensibly appear to challenge these very notions.” For Adkins (2004, p. 203), theorizing detraditionalization sits more convincingly within a Butlerian understanding of mimesis: that is, ambivalences at the core of mimesis play important roles in potentially interrupting hegemonically gendered norms. In Butlerian terms, gender is “an endless repetition of itself, a compulsive production or mimicry of its impossible” (Bell, 1999, p. 89). Gender can, therefore, be thought of as “a norm that can never be fully internalised, [gendered roles are] phantasmatic, impossible to embody…” (Butler, 1990, p. 179) insofar as “the addressee never quite inhabits the ideal/s s/he is compelled to approximate” (Butler, 1993, p. 231). In turn, mimesis can be thought of as dynamic and precarious. Adkins (2004, p. 202) argues that this understanding of mimesis provides a more plausible way to speak of the possibilities of interrupting norms embedded within traditional “arrangements of gender.”

It is within this understanding of mimesis that she returns to Bourdieu's theory of practice: that it is within the internal taking up of gendered norms by individuals, as part of the cultivation of their habitus that moments of instability—through ambivalence—arise. Adkins relies on Bourdieu's definition of practice as prereflexive and unconscious to illustrate how these norms are taken up by individuals. This process is based on what Adkins (2004, p. 206) calls a “logic of iteration” (p. 206) whereby the taking up of norms becomes a product of temporal performances, always adapting and accommodating to the moment. This logic, she (2004, p. 206) argues, “explains both how and why possibilities of instability, ambivalence and interruptability are at the core of mimesis and ambivalence.” Adkins (2004) then asserts that because norms are never fully incorporated (thus making the ideal unattainable), ambivalence is at the very core of mimesis (as prereflexive) and creates possibilities for gender detraditionalization (Adkins, 2004, p. 191). In this way, change is not conceptualized as external, but rather “internal to the operation of norms themselves” (Adkins, 2004, p. 206).

Therefore, as well as examining who has had authority over certain discourses of male partner violence at certain times in history, this article will adopt Adkins’ (2004) approach to theorizing gender detraditionalization to explore the often subtle ambivalences in language that appear when mapping how male partner violence has been understood and talked about over time. The ultimate aim of this work is to explore new ways of understanding how male partner violence could be destabilized.

## Mapping the Language of Male Partner Violence

This article is inspired by Wetherell and Potter's (1992) mapping of language to address social problems. Theirs is a map of the language of racism, whereby they charted the discursive terrain of the problem in order to both “chart” its “themes and ideologies” (Wetherell & Potter, 1992, p. 1) and to shed light on how racism could be destabilized in society. This type of mapping exercise helps researchers find their “way round a certain terrain” (Wetherell & Potter, 1992, p. 1). This article uses this approach to mapping language of male partner violence. Throughout the following sections, a chronological map showing how language has developed and changed will be charted. This map will then be used to analyze what these language patterns can tell us about male partner violence and the possibilities for disrupting its pervasive presence in society. Please note, the focus of this article is restricted to language patterns predominantly used within English-speaking countries.

It is commonly thought that the second wave of feminism, and in particular the Battered Women's Movement of the 1970s, brought a critical gendered understanding of male partner violence for the first time to the forefront of public debate. While this period in history was pivotal in redefining the problem (see Dobash & Dobash, 1979; Dobash et al., 1992; Pagelow, 1981), the parameters—or in other words the thinkability—of this form of gendered violence and it impacts on women were socially defined and addressed much earlier (Aitken, 2007; Amussen, 1994; Cyr, 2015; Dolan, 2003; Gordon, 1988; Hunt, 1992; Lentz, 1999; Moore, 2002; Peterson del Mar, 1996; Pleck, 1983, 2004; Schechter, 1982; Sewell, 1989; Tomes, 1978).

### A Husband's Right to Beat His Wife

Since as far back as the Roman Era, historians have noted the presence in society of men using violence toward their female romantic partners (Sewell, 1989). These premodern societies were firmly patriarchal, which “deemed a wife the property of her husband and therefore subject to his control” (Sewell, 1989, p. 985). The Christian Church was a key field that promoted this understanding of a husband's right to control his wife, even giving authority to husbands to sentence their wives to death for not showing Christian virtues of “docility, chastity and passivity” (Sewell, 1989, p. 986). In the 1400s, the Catholic text *Rules of Marriage* outlined a husband's right to “stand as judge of his wife and to beat her with a stick upon commission of an offense” (Sewell, 1989, p. 986). While the 1500s brought about new understandings of marriage as a relationship based on companionship rather than just male ownership, it was acceptable for husbands to use violence as a way to control their wives (Lentz, 1999).

Over the next three centuries (1500–1700s), key shifts occurred in how “wife-beating”—as it was commonly termed—was understood by society. The first key shift saw a public condemnation of husbands killing or using extreme forms of violence toward their wives. Due to a lack of any police infrastructure, historians note that neighbors would often intervene if a husband's use of violence was deemed extreme. This meant that these social fields—the community itself—contributed to a growing belief that it was unacceptable for men to use severe violence toward their wives (see Amussen, 1994). It should be noted though that these neighborly interventions only occurred among the common class due to these communities living in close confines to one another. Amussen (1994, p. 81) notes that the gentry class did not appear to experience these types of bystander involvements, making wealthier women more “vulnerable” to “wife-beating” at this time.

Other key fields—again the Church and the establishment of the judiciary (courts or magistrates)—also redefined a husband's right to use violence toward his wife. Various magistrates across both the United States and the United Kingdom were vocal in their disdain of “wife-beating,” with a number of courts (e.g., outlawing “wife-beating” at this time. For example, in the mid-1600s laws were passed in Massachusetts and New Plymouth Bay that made “wife-beating” unlawful (Sewell, 1989). Even though this changed public perception of “wife-beating,” courts rarely enforced punishments (Sewell, 1989). This was for a number of reasons: court proceedings and divorce applications, for one, were expensive; and even when cases were brought to court, magistrates tended to prefer families stayed intact (Hunt, 1992; Sewell, 1989). In a similar contradictory fashion, the Church at this time was also vocal against husbands using violence toward their wives, however many also prefaced this with circumstances where “wife-beating” was deemed an “allowable punishment” (Sewell, 1989, p. 987), for example, to keep an “orderly household” (Amussen, 1994, p. 77).

This exoneration of certain degrees of violence was further stabilized in the eighteenth century by key figures within the British judiciary. William Blackstone and Francis Buller both wrote on this topic, with Buller famously coining the phrase—*Rule of Thumb*—a standard of measurement that gave husbands the right to use violence toward their wives as long as any implement used was no thicker than his thumb. All of these legitimations of men's violence “served to strengthen the legal base of patriarchy” (Amussen, 1994, p. 83), which is turn “gave greater power to husbands” (Amussen, 1994, p. 72).

Therefore, while male partner violence did have a discursive reality at this time—in that it was thinkable with different fields in society making known its negative effects on women—it was dealt with in very inconsistent and contradictory ways with men having substantial freedom in their use of violence toward their wives (Lentz, 1999). One can assert that at the level of habitus, women's experiences of “wife-beating” and their ability to find support was complicated. While certain figures within the Church and the judiciary condemned this form of violence, finding help in the form of divorce proceedings or having their husbands punished was rare. Historians also note that women were more likely to be further disadvantaged if they did seek help. For example, Moore (2002) outlines how women would often experience extreme poverty if their husbands were either imprisoned or fined for their violent behavior. Because of this, many women chose either not to press charges or if they did were unable to keep bringing their recidivist husbands to justice.

### Misogyny, Paternalism, and First-Wave Feminism

Across the nineteenth century, more changes took place in how society addressed “wife-beating.” Across the UK and the US (the US took many of its cues during this time from English Common Law—see Sewell, 1989), two distinct and contradictory viewpoints about “wife-beating” surfaced. These viewpoints, for the most part, surfaced from judicial fields at the time.

The first viewpoint, principally stemming from the UK, was highly misogynistic in its evaluation of “wife-beating.” In his 1877 handbook *Principles of Punishment*, English lawyer Edward Cox wrote that most women deserved the violence they experienced from their husbands and that “unprovoked brutality and cruelty to helpless and submissive women” was rare (Tomes, 1978). As Tomes (1978, p. 339) notes, “many magistrates” adhered to this belief, which in turn led to a lack of punishments given to violent husbands. Fueling this viewpoint was an enduring support of the *Rule of Thumb*, that continued to dominate public understanding of how husbands could treat their wives. In the US, a number of states (namely Mississippi in 1824 and a collection of southern United States thereafter) even wrote the *Rule of Thumb* into law. It should be noted though that this was not universal across the US; courts in the more progressive state of California, for example, openly rejected the *Rule of Thumb* around the same time (Lentz, 1999).

The second viewpoint contested Cox's assertion, openly condemning this form of violence. In the UK, a dominant proponent of this was Thomas Holmes—a Police Court Missionary in London. He argued that men ought to be paternalistic toward their wives and, therefore, not use violence. According to Holmes, violence of this kind was “unmanly and cowardly” (Tomes, 1978, p. 339). Holmes’ view was profoundly classist in nature, however. It presumed that working-class men were the main perpetrators of violence toward wives. This classist belief was strengthened at this time in the UK (and in the US—see Peterson del Mar, 1996) as working-class men in general were seen as engendering brutish, “ruffian” and immoral qualities (Tomes, 1978, p. 339).

Holmes’ discourse influenced a number of judicial measures put in place to protect women from their violent husbands (Tomes, 1978); these changes however did not occur till the latter part of the 1800s. In the UK, an amendment to the Matrimonial Cause Act in 1878 made separation a legitimate avenue for women to pursue and in 1888 the Wifebeaters Act was passed outlawing this from of violence. Also at this time, The Society of the Protection of Women was set up in Britain with the mandate of addressing the welfare of women: “wife-beating” was one such issue that the Society addressed (Tomes, 1978). By the latter part of the 1800s, the US (and most other Western nations—Sewell, 1989) illegalized “wife-beating” including a widespread abolishment of Rule of Thumb clauses from law (Lentz, 1999; Sewell, 1989). Even with these important changes, “wife-beating” continued to endure. As Cyr (2015) explains, even with new legislation, there was an overarching tolerance of “wife-beating” across the west, which meant that many men were never prosecuted.

While the US followed much of English Common Law during this century (Sewell, 1989), it is important to note that settler gender politics meant there were additional variations in how “wife-whipping”—as it became commonly termed in the US (Peterson del Mar, 1996)—was dealt with at this time. Settler men principally showed their manliness through being self-disciplined and self-sustaining (rather than paternalistic, as promoted by Holmes-esque discourses in the UK). These virtues, however, were highly contradictory when it came to social attitudes toward “wife-whipping.” For example, within settler communities, giving into violent rages was seen as the antithesis of how a self-disciplined husband should act. In contradiction, however, the masculine virtue of self-discipline and sustainability made violence of this kind, at times, acceptable as it aligned in many ways with a husband's duty to protect and discipline those dependent on him. To further complicate how “wife-whipping” was understood in these settler communities, manliness was also associated with being rugged and tough “frontiersmen” (Gordon, 1988, p. 255). This construction gave men some added freedom to control their wives using violence. As Peterson del Mar (1996, p. 10) notes, “male violence commonly served as a tool to enforce male authority.”

The Church through-out the west, although still a powerful field at this time, appeared to play less of a role in historical accounts of how “wife-beating” was addressed in the 1800s. This may have been due to the privatization of religion during this century alongside the increased work courts (that were now increasingly secular) were carrying out to address this form of violence (Ruether, 1984). In saying this, however, the Church and its doctrines regarding divorce did influence discursive constructions of “wife-beating” during the 1800s. Therefore, while access to divorce was easier for women in the 1800s (Lentz, 1999), the Church while largely opposing “wife-beating” were also vocal in their disapproval of divorce. Therefore, “women's flight from the violence” (Peterson del Mar, 1996, p. 44) was not condoned. For example, in his 1880 *Sermon to Women* Reverend Knox-Little asserted that divorce was never justified, even “if [the husband] be a bad or wicked man” (Gage, 1893, p. 493).

Even though women at this time were largely seen and treated as passive to men, this did not deter many from resisting dominant discourses surrounding men's privilege and ownership over women (Sewell, 1989). Prominently, this resistance was seen in the activities of first wave of feminism that swept across the western world from around the mid-1800s. In 1848, feminist reformers in the US held the Seneca Falls Convention, which was the first convention to address women's rights (Lentz, 1999). Here, the *Declaration of Sentiments* (Stanton, 1848)—outlining the ways in which women had been oppressed by men and a declaration of intent for emancipating women from patriarchal rule—was presented and signed by attendees of the conference. As the nineteenth century progressed, feminist voices continued to grow across the UK, the US, and other western countries.

Temperance and suffrage were the most popularly recognized issues that this first wave of feminism tackled. More taboo issues, like violence in the home, tended to be addressed within broader discussions around legal rights of women (Aitken, 2007; Gordon, 1988; Lentz, 1999; Pleck, 1983; Sewell, 1989). Feminists were not naïve though, to think that elevating women's legal status would automatically protect women from “wife-beating.” In fact, many acknowledged that giving more rights to women may increase men's use of violence as a form of control (Lentz, 1999). The main messages feminists brought to the public arena regarding “wife-beating” were two-fold: one, that women needed more protection from their violent husbands (feminists tended to see working-class women as most vulnerable (Aitken, 2007; Lentz, 1999); and two, that this form of violence was unequivocally caused by the “rule of men” (Lentz, 1999, p. 19). Here, a feminist critique of the patriarchy in relation to men's ability to use violence toward women was now established.

Even with this broad consensus, different feminist groups addressed “wife-beating” in different ways during this era. One prominent group of feminists active at this time came from the emerging “bourgeois culture” in the west (Peterson del Mar, 1996, p. 47). These conservative feminists created a platform to address issues around violence prevention mainly through their work in setting up protection agencies for women and children. However, historians note that this group was *more* active in bringing violence toward children to a public platform than an awareness of “wife-beating” (Gordon, 1988; Pleck, 1983). This is not to say that women's rights were largely ignored by this group. Because these feminists were predominantly active members of the Protestant Church, their overarching opposition to divorce led them to support new legislation and policies to protect women from male partner violence; the aim here was to make marriage more tolerable for women (Gordon, 1988; Pleck, 1983).

Another feminist organization active throughout most of the western world at this time was the Women's Christian Temperance Union (WCTU) (Aitken, 2007). WCTU members also tended to be conservative, wealthy women and as the name suggests, their focus was mainly on men's consumption of alcohol (Gordon, 1988; Pleck, 1983). The WCTU addressed “wife-beating” by arguing that men's use of alcohol was the mitigating factor (Aitken, 2007; Gordon, 1988; Pleck, 1983). This approach tended to frame “wife-beating” as an individualized problem driven by men's pathology, which in turn silenced more critical discussions around gendered societal power. In addition to temperance, the WCTU—like their other feminist counterparts involved in protection agencies—also reflected the protestant–conservative discourse surrounding marriage. They voiced their disapproval of divorce and saw the preservation of marriage as an important moral standpoint even when male partner violence was present (Gordon, 1988; Pleck, 1983).

Liberal feminists were also active at this time and appeared to have a more woman-focussed agenda. Unlike their protestant–conservative counterparts, liberal feminists lobbied in favor of divorce in order to help wives leave violent husbands. This was in line with their liberal agenda to address inequality amongst men and women. Their emphasis on divorce, however, meant that their discussions surrounding “wife-beating” and its detrimental effects on women were more peripheral and never gained significant momentum during their active years (Pleck, 1983).

These new authorized voices stemming from the field of first-wave feminism all contributed to a more critically gendered thinkability of “wife-beating” at this time, which would have made it easier for women experiencing “wife-beating” to find support (Gordon, 1988). Feminist discourses, however, still had to compete with nonfeminist voices at this time. As has been outlined, competing discourses surrounding “wife-beating” were either paternalistic, casting women as “passive creatures” who needed men to protect them (Tomes, 1978, p. 342) or were more overtly misogynistic, seeing women as pariah-like “fallen angels” (Tomes, 1978, p. 339) who were both aggressive and deserving of men's violence. Therefore, at the level of habitus, women's ability to make sense of the violence they were experiencing and even being able to find avenues of support would have been complicated by the many conflicting ways in which “wife-beating” was understood at the time (Aitken, 2007; Gordon, 1988; Lentz, 1999; Peterson del Mar, 1992).

### An Era of Silence

This era of feminist debate and awareness of male partner violence was not sustained (Gordon, 1988; Pleck, 1983), which aligned with an organizational shift within the very protection agencies that conservative feminists had set up. From the early 1900s, feminists were replaced by a new wave of middle-class professionals who introduced what was seen by many as a more “scientific” management-style approach to running these agencies (Gordon, 1988, p. 62). This new field of social services was, therefore, seen as a more efficient in comparison to how conservative feminists managed these agencies up until this point.

These new “professional” social agencies became the authority on all matters pertaining to the family unit (Gordon, 1988). One characteristic of this new field was the introduction of case work reports, which quickly became a way to surveil families who were seen to come from the underclass (largely made up of immigrant communities) in society (Gordon, 1988). A key thread throughout their rhetoric was the notion of the “undeserving poor,” which largely targeted mothers. This was because, more so than in previous years, child neglect was now discursively attached to the notion of the transgressive and deviant mother (Gordon, 1988). This misogynistic focus meant that many women experiencing male partner violence were unable to seek help for to fear of being prosecuted themselves or losing custody of their children (Gordon, 1988).

This focus away from feminist discourses of male partner violence was also led by a shift in what constituted male privilege in the home. While the early 1900s saw a brief emergence of a “new sensibility of domesticity” (Gordon, 1988, p. 41), which included a promotion of the *gentle*-man—praised for being a devoted but detached family man (Gordon, 1988; Peterson del Mar, 1996), a more controlling version of male privilege took hold across the 1920s and 1930s. Men were no longer praised for being gentlemen in their families; instead, men were encouraged to exert more control and authority over their wives (Gordon, 1988). This newfound right for men to be the authority over their families was further entrenched by families becoming more ameliorated as urbanization and the privileging of the nuclear family took hold throughout western countries. (Peterson del Mar, 1996). This shift to a more individualized way of life meant that many women did not have the same community ties and social bonds with other women, which in turn created an environment whereby male partner violence became a private matter (Peterson del Mar, 1996).

Divorce was still a difficult option for women to contemplate. The church at this time still condemned divorce and the misogynism that was building in the social services field meant that women who did leave violent marriages also risked being positioned as transgressive, immoral, and to blame for any “dysfunction” in their family unit. Gordon (1988) outlines the following struggles these women faced within the early decades of the twentieth century:

Nowhere was the tension between social work standards and values, and client's needs and possibilities, greater than in the case of single mother families. Single mothers were the most wretched group of clients. This was partly because they were the poorest but also because they were in a double bind as they struggled to meet the contradictory expectations of raising and providing for children in a society organised on the premise of male breadwinning and female domesticity. (Gordon, 1988, p. 83)

This would have resulted in many women enduring a conflicted, confusing and fear-filled engagement with habitus at this time.

### Women to Blame

In keeping with this developing narrative of transgressive women, the period between World War II and the 1960s saw a more sustained period of misogynism take hold across the global west. Not only confined to the field of social services, this woman-blaming discourse became widespread across other fields of action. As Schechter (1982, p. 27) remarks, “the police, courts, hospitals, and social service agencies cooperated, although not conspiratorially, in defining the abuser's behavior as legitimate or insignificant and the victim's behavior as crazy, provocative, or reformable.” This new way of making sense of male partner violence was particularly manufactured by psychiatrists and psychologists who authored a range of journal articles on the matter (see Faulk, 1974; Lion, 1977; Shainess, 1977; Snell et al., 1964). These medical professionals made up a new dominant field of medicine that now claimed authority on issues relating to the family.

This field shifted how society understood male partner violence in a number of ways that had detrimental consequences on women at the time. How male partner violence was labeled, was one of these ways. Language used to describe male partner violence now became euphemized by medical professionals (see more Gordon, 1988, p 284) with lexical arrangements like “marital discord” (see Snell et al., 1964) and “marital dysfunction” (see Shainess, 1977) becoming commonly used. These terms not only denied the gravity of male partner violence but also erased the “doer” of this form of violence from medical discourses relating to this problem.

This euphemistic treatment of male partner violence by the medical field also coincided with a promotion of a suite of misogynistic narratives relating to the problem. While many medical professionals (Faulk, 1974; Lion, 1977; Snell et al., 1964) agreed that anger manifested within men who were violent toward their wives, it was largely suggested that these men were provoked into being violent by their masculine, aggressive, and/or controlling wives. Thus, it was suggested that women brought “discord” into the home while men simply reacted to it (Snell et al., 1964). As Faulk (1974) argued, a wife's provocation (characteristics of what he termed “a quarrelsome wife”) was a dominant reason why a “dependent passive husband” (p. 121) became violent. Lion (1977, p. 129) also drew on the notion of provocation: “The phenomenology of wife-battering involves the victim playing a major role. Recognition of this is mandatory, and should not be seen as pejorative…the issues involve the ambivalences and pathologies of both partners.” Shainess (1977) argued specifically that a woman's predisposition to premenstrual mood imbalances was difficult for husbands to manage and could provoke frustration and anger. Shainess, therefore, recommended wives ascertain the role they played in causing the violence in order to correct their behavior and prevent the violence from happening again.

Women were also blamed for the violence they were experiencing in the home because of what many doctors deemed to be their sexual frigidity. Shainess (1977, pp. 117–118) recommended: “If the husband can be considered to be ‘sexually addicted’ or compulsive in his need for sex, then while not desirable for his wife, nevertheless, if she desires the marriage to continue, it makes for a more tolerable marriage to be compliant…if she wants the marriage to continue, she is the one who must make the greatest effort” (pp. 117–118). Social workers at the time also singled out women's frigidity as a root cause of men's violence. This was a view very different from the earlier Victorian days where social workers often supported wives who had a sexually demanding and violent husband (Gordon, 1988). Some medical professionals also maintained that women *wanted* to be mistreated by their romantic partners (Lion, 1977; Snell et al., 1964). As Lion (1977) proposed: “some women fear kind husbands and gentle men” (p. 132) … [while] some women need someone who they can feel sorry for “as a defence to protect her from her own anger and assertiveness” (p. 133).

The medical field also perpetuated explanations for why husbands used violence. Largely it was argued that a husband's actions were due to pathological forces external to his control (O’Neill, 1998). For example, Faulk (1974) surmised that men were most likely to be violent because of stress. The other predominant rationale for a husband to be violent, in Faulk's assessment, was mental illness. These pathological frames worked to exonerate violent men, and in turn also fed into an individualistic discourse that would span the next 100 years: if men could not help being violent, then in was up to the women to solve the problem. This made women's behavior change the focus of medical recommendations for treating the problem and in turn formed the basis of an enduring narrative—that women are culpable for staying in violent marriages (O’Neill, 1998).

Historians note that during this period of medicalized woman-blaming, male partner violence increased in frequency while also being treated largely as a private matter (Peterson del Mar, 1996). Women, therefore, faced huge difficulty getting support that could actually keep them safe. At the level of habitus, men were enabled to be violent within their romantic relationships yet women who spoke out or sought help from doctors, were vilified. In this way, male partner violence has less of a discursive reality than in previous eras and therefore, became primarily “unthinkable” during this time.

### Feminist Rediscovery

This period of unthinkability was not sustained; by the 1970s a groundswell of second-wave feminism—and the emergence of the Battered Women's Movement—created an important platform for reintroducing critical gendered definitions of male partner violence into public debate. Feminism, at this time, had a lot of work to do unraveling the misogynism embedded in definitions of male partner violence from the previous three decades. This work was started by feminists involved to the Women's Liberation Movement: a sub-set of feminists (mostly from radical and socialist feminist groups) who not only fought for women's rights to equal voice but carried out a strong political agenda that addressed gendered roles and power in society. As a political movement, it did more than *just* address equality; it created a platform to publicly address the privatized experiences of women that were oppressive: namely women's denial of rights over their own bodies and women's experiences of motherhood, domesticity, and violence. This political work was pivotal in reintroducing male partner violence as a discursive reality (Schechter, 1982).

One of the key challenges the field of feminism at this time faced in the campaign against male partner violence was around changing the way the problem was talked about and therefore, understood. As feminist scholar Kersti Yllo (2005) comments, “the most fundamental feminist insight into all of this is quite simple: domestic violence cannot be adequately understood unless gender and power are taken into account” (p. 19). Michele Bograd (1988, pp. 13–14) broke this down into more detail:

Four major dimensions are common to all feminist perspectives on wife abuse: (1). The explanatory utility of the constructs of gender and power; (2). The analysis of the family as a historically situated social institution; (3). The crucial important of understanding and validating women's experiences; (4). Employing scholarship *for* women.

The first task feminists tackled was to shift public thinking from how male partner violence was currently understood—that this type of violence was a private marital matter usually caused by women's behavior. As Loseke et al. (2005, p. x, *emphasis added*) outline, “encouraging the general public to condemn violence therefore required changing the idea that violence inside homes was ‘normal’ and inconsequential. One mechanism for changing these attitudes was to *name the problem*” in ways that no longer de-validated women's experiences (see also, Schechter, 1982).

Terms like “wife-beating” and “wife abuse” (Yllo, 1988) and the more inclusive term “woman battering” (Pagelow, 1981; Schechter, 1982) were (re)introduced by feminists with the endeavor to discursively relocate violence in relationships within a gendered context: that is, with women's experiences of men's violence *as a gendered phenomenon* at the core of its understanding (Sev’er, 2002). In doing so, these terms also counteracted woman-blaming discourses of the previous eras by placing the “doers” of this gendered violence—men—as key to how the problem was understood. There were variations in terminology. These renamings and redefinitions of male partner violence by this feminist movement helped transform this form of violence from a “subject of public shame and misery to an object of public concern” (Tierney, 1982, p. 210).

Coinciding with feminists’ renamings of the problem was their role in the development and setting up of women's shelters. While there were shelter-like places made available to women during the first wave of feminism (Gordon, 1988; Pleck, 1983), the shelters of the 1970s were set up in far greater numbers and were more *active* in promoting feminist ideologies. These shelters were originally spaces for women to collectively meet and discuss a wide-range of subjects relating to their gendered experiences; in time, these spaces became specifically sites of refuge for women from violence as this was what women primarily wanted to talk about. Here, a politics of talk was emerging within shelters, which in turn meant that terms like the “battered woman” started to become publicly recognized (Schechter, 1988). This had great benefits for women: at the level of habitus, the victim-blaming language of the previous era was now being challenged, which in turn provided women with the ability to speak out, collectively, making shelters a key location for the “personal is political” discourse of the 1970s to develop (see Ahrens, 1980; Ferraro, 1983; Schechter, 1982, 1988).

Feminist scholarship concerning male partner violence prevention also emerged at this time, with a growing number of feminist researchers vocalizing a critical gendered discourse in addressing the problem (Dobash & Dobash, 1979; Pagelow, 1981; Schechter, 1982; Yllo, 1988). As discussed previously, the authorities of the 1930s–1960s were influenced by misogynistic research conducted within medical and psychology disciplines (Faulk, 1974; Lion, 1977; Shainess, 1977; Snell, Rosenwald & Robey, 1964). Feminist researchers, therefore, tasked themselves to confront and dispel the layers of misogyny that founded these “myths” (see Pagelow, 1981). Stark et al. (1979, p. 463), for example, presented a valuable critique of medical reporting, which they argued promoted “female brutalization” in the ways in which women experiencing male partner violence were pathologized as being mentally unwell. Chesler (1971, p. 746) extended this critique to argue that medicalized knowledge prevented women's ability to seek appropriate help and support due to the ways in which medical professionals presented themselves as “benevolent authorities” in women's lives: “That both psychotherapy and marriage, the two major socially approved institutions for white middleclass women, function similarly, ie. as vehicles for personal ‘salvation’ through the presence of an understanding and benevolent (male) authority.”

Feminist researchers were also vocal against medical discourses that positioned women as to blame for their experiences of male partner violence. Dobash and Dobash (1979) described the climate of this discourse in the 1970s: “The idea that nagging provokes or causes the violence is not merely a justification provided by the man for his behaviour, it is very much a part of our cultural beliefs and is also used by researchers and representatives of social agencies to explain ‘wife-beating’ or to justify their own actions or inactions” (p. 133). Another discourse emerging at this time blamed women who chose to stay in violent relationships, placing the responsibility for ending the violence with women. Feminist researchers counteracted this victim-blaming discourse by breaking down the complexities of why some women stay and why some women leave violent male partners. As Schechter (1982, p. 20) lamented:

They forget that women weigh their alternatives and constantly make choices; any woman might decide to stay after asking herself, “What is it like to be beaten twice a year compared to living alone with two children, penniless?” If aid is offered and turned down, friends are angry; few are able to understand or feel sympathy for the woman who is ambivalent about leaving her husband. When people do not know what to do or lack resources to meaningful assist, their helplessness may turn to anger, not always at the circumstances, but at the person requesting help. They cope with this anger by blaming the victim.

This feminist critique confronted the masochistic narratives embedded in medical discourse: that if women stay in violent relationships, they must have a desire to experience violence (see Bograd, 1988; Pagelow, 1981; Schechter, 1982)—a “willing sufferer” (Pagelow, 1981, p. 55).

One key outcome of feminist reauthorizing of male partner violence across the 1970s and 1980s was the emergence of a more inclusive *and* gendered global definition of the problem (Abrar et al., 2000). On the global stage, this was marked by the UN Commission on the Status of Women, in 1992, formally defining all violence against women as “any act, omission, controlling behaviour or threat, in any sphere, which results in or is likely to result in physical, sexual or psychological injury to women” (Marcus, 1994, p. 30). Here, a formal recognition of *gender-based* violence that need not only be physical, was declared on the global stage (Marcus, 1994).

This widening of the definition of male partner violence coincided with the introduction of a new theoretical paradigm, also informed by feminist activity of the time, that would prove to be influential in reframing how male partner violence is understood (Dobash & Dobash, 1992). This paradigm, known commonly as the Power and Control Wheel, was devised by the Duluth Domestic Abuse Intervention Project in the 1980s—a ground-breaking project that shifted the focus from woman-blaming rhetoric to bettering legal and judicial responses to prosecuting and rehabilitating violent men that would have widespread take-up across the world (Dobash & Dobash, 1992). The Wheel itself has been pivotal in a number of ways: it is unambiguously gendered, which adds clarity around what this type of violence actually looks like and it recognizes psychological and other nonphysical coercions as part of this form of violence that women experience. It is still used widely today.

At this time, legislation was also being introduced in many countries recognizing this full suite of violence—physical, psychological, and sexual—women face in their romantic relationships (Ortiz-Barreda & Vives-Cases, 2013). In Aotearoa/New Zealand, for example, the Domestic Violence Act was passed in the mid-1990s that added some definitional clarity around the problem: According to this Act, “domestic violence is any physical, sexual and psychological abuse, the last of which includes, but is not limited to intimidation, harassment, damage to property, threats of violence and committing acts of violence in front of children” (Law Commission, 2001, p. 2). Prior to this, violence in the home was still largely considered a private matter and while there was an earlier piece of legislation in New Zealand that did have some protection measures in place for violence in the home, the Domestic Violent Act 1995 had a more targeted and definable set of principles in place as well as clearer and streamlined procedures like compulsory police reporting and greater ease of applying for protection orders. The 1995 Act also recognized that nonmarried couples were now covered by the law, including homosexual couples.

All of these discursive shifts across the 1970s and 1980s meant that at the level of habitus, women's rights to live free from male partner violence was again “thinkable” largely due to the ways in which feminist voices authorized this new way of viewing the problem. In turn, women were now able to access more appropriate support regarding the violence they were experiencing that women's experiences of domestic life too shifted. No longer was male partner violence *just* a private marital affair that “good” wives had to endure and importantly, marriages now were relationships that women could more easily leave. Women were also more able to find avenues for support from a wider variety of fields: the police, lawyers, and other social service providers, like women's shelters and other violence prevention advocacy agencies.

### A Gender-Symmetric Shift

While the field of second-wave feminism made significant advances in how male partner violence was discursively understood from the 1970s onwards, competing nonfeminist voices emerged that shifted how many in society made sense of the problem. One reason for this shift occurred within the shelter movement. From the 1980s onwards, the running of shelters was taken over by the field of social services. This predominantly nonfeminist field introduced a management culture into how shelters were organized and run. This shift meant that shelters took on corporate-like hierarchies, with women seen now as clients rather than participants within a feminist nonhierarchical space (Ahrens, 1980; Ferraro, 1983; Schechter, 1988). These factors, combined, led to the return of individualized, therapy-driven strategies for dealing with the problem, while also positioning women as dependent subjects, in need of treatment (Ahrens, 1980; Ferraro, 1983). Shelters also became locations for *anti*-feminist discourses to gain momentum. For example, during the 1980s, the rift between feminist- and nonfeminist-run shelters occurred as a narrative around “dangerous lesbianism” emerged; here, feminist-run shelters were seen as being led by lesbian radicals whose intentions were to destroy marriages by turning straight women gay (Ahrens, 1980; Schechter, 1988).

There has also been a growing focus on defining this form of violence from nonfeminist viewpoints across academic departments like psychology and sociology (Bograd, 1988). Most notably, it has been within the field of sociology and specifically the development of the Conflict Tactics Scale (CTS) by family sociologist Murray Straus (1979) that has caused marked changes in how society made sense of and talked about male partner violence (Dutton & Bodnarchuk, 2005; Gelles, 1985; Straus, 1979, 2005).

Still active in this research today, Straus and colleagues predominantly look at how families experience and manage conflict. The CTS is a quantitative, survey data-gathering method designed to empirically account for any conflict that research participants have experienced in their domestic environments. This method is still one of the most widely used tools in family violence research, due to its simplistic, quantitative features (Langhinrichsen-Rohling et al., 1995). From their findings, Straus and colleagues concluded that violence within families was gender-symmetric: that both men and women were equally violent in these spaces.

Feminists have widely critiqued this method of inquiry arguing that the scale itself provides little context about the violent occurrences being reported by its participants (DeKeseredy & Schwartz, 1998; Dobash & Dobash, 1979; Yllo, 2005). As DeKeseredy and Schwartz (1998, pp. 3–4) argue:

The bulk of the research in this field has simply counted blows (who hit whom, and how often). The CTS2 speaks to one context issue (but only one) by asking about injury. A light slap may be different than one that jars loose several teeth. A push out of the way is different than a push down a flight of stairs. However, the survey still does not easily differentiate between a victim fighting back for her life, a survivor retaliating, and an instigator of violence without cause. All are considered violent.

Accordingly, this has silenced the reality that men use more severe forms of violence in romantic relationships than women do and that women experience higher degrees of fear from a romantic partner than men do (DeKeseredy & Schwartz, 1998; Dobash & Dobash, 2004; Sev’er, 2002). The scale also does not differentiate from self-defense and other forms of physical violence, meaning that any act of self-defense (largely carried out by women: see Law Commission, 2001) is classed simply as physical violence. Overall, the CTS fails to account for gendered components of power and control, instead focussing more on argument–disagreement types of conflict (DeKeseredy & Schwartz, 1998; Dobash & Dobash, 2004; Sev’er, 2002).

Regardless of these critiques, the gender-neutral discourse created by Straus and his colleagues has continued, up until this day, to perpetuate an understanding in society that partner violence is *not gendered.* Arguably, this understanding has been further cemented by the way that legislation in western world is worded: typically legislation and policy documents engage gender-neutral narratives by using terms like “domestic violence” or “family violence.” These labels can silence—or make unthinkable—the uniquely gendered experiences of male partner violence by clumping all types of familial violence—be it toward children, siblings, elders, or partners—together. Male partner violence, therefore, becomes lost in these catch-all gender-symmetric discourses. Today, in Aotearoa/New Zealand, the key legislation protecting women from violent partners is the 2019 Family Violence Act. Sadly, it falls, discursively, into the pitfalls of gender-symmetric understandings of violence in the home. It is ambiguous—it does not acknowledge that the violence women experience from a male partner is gendered, it does not differentiate adequately between this type of violence and say child abuse, it muddies the lexical terrain by using the word “family” which means that nonmarried and noncohabiting relationships are less recognized in the law. Arguably the term “domestic violence” is clearer than “family violence” when it comes to women's experiences of a violent male partner. Indeed, earlier terms that seem archaic now—“wife-beating” and “woman battering”—were more nuanced in gendering the problem.

While there is a range of gendered spaces and responses dedicated to helping women—for example, women's shelters and other advocacy organizations, streamlined legislation and models like the Power and Control Wheel that enable women to access more support than say in previous decades—gender neutral discourses *do* impact how the problem is understood and talked about in society. Due to these current discursive ambiguities and contradictions, women who experience male partner violence are again navigating a habitus that is likely not well-defined and potentially confusing. This contradictory discursive space also means that men are enabled to be *more* violent because women's agency is yet again hampered by dominant fields of action authorizing knowledge that preserves male privilege.

## Destabilizing Male Partner Violence

The article has mapped the various ways language had addressed the problem of male partner violence. Throughout this mapping exercise, three patterns have emerged. One, men using violence in their (hetero)romantic relationships has been a consistent part of society for a very long time. Two, even during times when women-focussed discourses have surfaced (e.g., during the first and second wave of feminism), this gendered awareness has continued to be overshadowed and silenced by contradictory discourses that detract from women's experiences of violent male partners. Three, there has been no time in history when cogent and consistent language has been widely adopted to fully address women's experiences of this gendered form of violence (Easteal et al., 2012; Marcus, 1994; Minow, 1990)

What can these historical patterns, therefore, teach us about the problem of male partner violence? Most noticeably, they tell us that there is a supremacy of patriarchal power and privilege that enables male partner violence to continually endure. These patterns also tell us that language matters. If popular and widespread gender-neutralizing and nonfeminist terms like “family violence” and “domestic violence” were replaced by the term “male partner violence” in a range of fields—government and courts (legislation and policy documents), the media, schools, the church, health, and wellbeing providers—then a shift at the level of habitus would likely occur. People would have more dispositions to draw on that “have the effect of calling into question rules of dominant discourse for forming statements” (Alcoff & Gray, 1993, p. 268) about this form of gendered violence. This, in turn, has the potential of changing the landscape of how male partner violence is addressed in society, making violent male partners more accountable and women experiencing this form of violence more supported.

These historical patterns also teach us more subtle lessons about male partner violence. Arguably, these less obvious lessons are particularly important to consider when a problem—like male partner violence—is so ubiquitous. By historically mapping how male partner violence has been talked about and understood over time, what becomes apparent is that the language used to describe the problem—and therefore the incorporation of social norms surrounding how to make sense of and act with regard to the problem—has never been static or fixed. This constant state of flux suggests that fragility of patriarchal power has consistently been present throughout history. By adopting Adkins’ (2004) feminist Bourdieusian framework, this fragility of patriarchal power, and the possibilities this shows about how this male partner violence could be destabilized, can be critically examined in new ways.

Adkins framework helps to explain how an inconsistency in how male partner violence has been understood and talked about over time could signal a fragility within patriarchal power itself. To summarize, from historically mapping the language used to discuss this form of violence, patriarchal structures have imbued fields like the Church, the medical world, the social services sector, and quantitative research groups with a range of normative meanings about the problem. For example, that violence of this kind is largely women's fault, that it is a private matter, and that it is not based on gender. Therefore, one's ability to make sense of male partner violence has been impacted by these normative meanings. However, unlike other forms of violence that have enjoyed quite stable definitions—for example, male–male assaults/fights—language used to discuss male partner violence has been significantly inconsistent and ever-changing. This in turn has created degrees of ambivalence when the problem is discussed.

If society cannot settle on a cogent language to address male partner violence, then I suggest that this ambivalence signals “cracks and fissures” (Jackson and Salisbury, 1996, p. 107) within patriarchal power *as well as* within people's internal taking up of “norms” relating to the problem (Adkins, 2004, p. 206). Therefore, what this article proposes is that discursively, a patriarchal, nonfeminist understanding of male partner violence is *both* enduring and fragile, which in turn allows us to see disorganization within the very discursive construction of how male partner violence is made sense of in the social world.

## Final Thoughts

While this article does not attempt to remedy the problem itself, its aim is to start a dialogue about the more subtle weak points within patriarchal power structures and the ways in which language could be used—in ways not already thought of—to create new avenues to better the lives of women experiencing male partner violence. As mentioned, because male partner violence has endured such a ubiquitous state across history, exploring these less obvious ways of looking at the problem—for instance, within the subtleties of ambivalence—may shed new light on how to destabilize the problem. Moving forward, further research needs to look at the ways in which these ambivalences can create change, both within the fabric of society that enables men to be violent and within the lives of women who experience this problem in their daily lives.

## References

[bibr1-10778012231192571] Abrar S. Lovenduski J. Margetts H. (2000). Feminist ideas and domestic violence policy change. Political Studies*,* 48(2), 239–262. 10.1111/1467-9248.00258 18286750

[bibr2-10778012231192571] Adkins L. (2004). Reflexivity: Freedom or habit of gender. Sociological Review*,* 52(Suppl. S2), 191–210. 10.1177/0263276403206002

[bibr3-10778012231192571] Ahrens L. (1980). Battered women’s refuges. Feminist cooperatives vs. Social service institutions. Radical America, 14(3), 41–47.

[bibr4-10778012231192571] Aitken J. (2007). “The horrors of matrimony among the masses”: Feminist representations of wife beating in England and Australia 1870-1914. Journal of Women’s History*,* 19(4), 107–131. 10.1353/jowh.2007.0072

[bibr5-10778012231192571] Alcoff L. Gray L. (1993). Survivor discourse: Transgression or recuperation?. Signs, 18(2), 260–290.

[bibr6-10778012231192571] Amussen S. D. (1994). “Being stirred to much unquietness”: Violence and domestic violence in early modern England. Journal of Women’s History*,* 6(2), 70–89. 10.1353/jowh.2010.0321

[bibr7-10778012231192571] Bell V. (1999). Feminist imagination: Genealogies in feminist theory. Sage Publications.

[bibr8-10778012231192571] Bograd M. (1988). Feminist perspectives on wife abuse. In Yllo K. Bograd M. (Eds.), Feminist perspectives on wife abuse (pp. 11–27). Sage Publications.

[bibr9-10778012231192571] Bourdieu P. (1977). Outline of a theory or practice (R. Nice Trans.). Cambridge University Press.

[bibr10-10778012231192571] Bourdieu P. (1984). Distinction: A social critique of the judgment of taste (R. Nice Trans.). Harvard University Press.

[bibr11-10778012231192571] Bourdieu P. (1990). The logic of practice. Polity Press.

[bibr12-10778012231192571] Bourdieu P. Wacquant L. (1992). An invitation to reflexive sociology. Polity Press.

[bibr13-10778012231192571] Butler J. (1990). Gender trouble: Feminism and the subversion of identity. Routledge.

[bibr14-10778012231192571] Butler J. (1993). Bodies that matter: On the discursive limits of “sex”. Routledge.

[bibr15-10778012231192571] Chesler P. (1971). Women as psychiatric and psychotherapeutic patients. Journal of Marriage and the Family, 746–759. 10.2307/349448

[bibr16-10778012231192571] Cyr H. D. (2015). A history of violence against women: Wife beating in the 19th century in America and England (Publication No. 4605874) [Master's thesis]. Southern Connecticut State University New Haven, Southern Connecticut State University ProQuest Dissertations Publishing.

[bibr17-10778012231192571] DeKeseredy W. S. Schwartz M. D. (1998). Measuring the extent of woman abuse in intimate heterosexual relationships: A critique of the Conflict Tactics Scales*.* http://www.vawnet.org

[bibr18-10778012231192571] Dobash E. Dobash R. (1992). Women, violence and social change. Routledge.

[bibr19-10778012231192571] Dobash R. E. Dobash R. (1979). Violence against wives: A case against the patriarchy*.* The Free Press.

[bibr20-10778012231192571] Dobash R. P. Dobash R. E. (2004). Women’s violence to men in intimate relationships: Working on a puzzle. British Journal of Criminology, 44(3), 324–349. 10.1093/bjc/azh026

[bibr21-10778012231192571] Dobash R. P. Dobash R. E. Wilson M. Daly M. (1992). The myth of sexual symmetry in marital violence. Social Problems, 39(1), 71–91. 10.2307/3096914

[bibr22-10778012231192571] Dolan F. E. (2003). Battered women, petty traitors and the legacy of coverture. Feminist Studies, 29(2), 249–278. https://www.jstor.org/stable/3178509

[bibr23-10778012231192571] Dutton D. G. Bodnarchuk M. (2005). Through a psychological lens: Personality disorder and spouse assault. In Loseke D. R. Gelles R. J. Cavanaugh M. M. (Eds.), Current controversies on family violence (pp. 5–18). Sage Publications.

[bibr24-10778012231192571] Easteal P. Bartels L. Bradford S. (2012). Language, gender and ‘reality’: Violence against women. International Journal of Law, Crime and Justice, 40(4), 324–337. 10.1016/j.ijlcj.2012.05.001

[bibr25-10778012231192571] Fairclough N. (2013). Critical discourse analysis: The critical study of language*.* Routledge.

[bibr26-10778012231192571] Faulk M. (1974). Men who assault their wives. Medicine, Science and the Law, 14(3): 180–183. 10.1177/002580247401400307 4475341

[bibr27-10778012231192571] Ferraro K. (1983). Negotiating trouble in a battered women’s shelter. Journal of Contemporary Ethnography*,* 12(3): 287–306. 10.1177/0098303983012003004

[bibr28-10778012231192571] Foucault M. (1977). Power/knowledge. Pantheon Books.

[bibr29-10778012231192571] Gage M. J. (1893). Woman, church and state: A historical account of the status of woman through the Christian ages*.* Truth Seeker Company.

[bibr30-10778012231192571] Gelles R. (1985). Family violence. Annual Review of Sociology, 11(1), 347–367. 10.1146/annurev.so.11.080185.002023

[bibr31-10778012231192571] Gordon L. (1988). Heroes of their own lives: The politics and history of family violence*.* University of Illinois Press.

[bibr32-10778012231192571] Hunt M. (1992). Wife beating, domesticity and women’s independence in eighteenth-century London. Gender & History*,* 4(1), 10–33. 10.1111/j.1468-0424.1992.tb00143.x

[bibr33-10778012231192571] Jackson D. Salisbury J. (1996). Why should secondary schools take working with boys seriously? Gender and Education*,* 8(1), 103–115. 10.1080/713668477

[bibr34-10778012231192571] Joas H. Knobl W . (2011). Between structuralism and theory of practice: The cultural sociology of Pierre Bourdieu. In Susen, S. Turner B. S. (Eds.), The legacy of Pierre Bourdieu: Critical essays (A. Skinner Trans.) (pp. 1–32). Anthem Press.

[bibr35-10778012231192571] Langhinrichsen-Rohling J. Neidig P. Thorn G. (1995). Violent marriages: Gender differences in levels of current violence and past abuse. Journal of Family Violence, 10(2), 159–176. 10.1007/BF02110598

[bibr36-10778012231192571] Law Commission. (2001). Report 73: Some criminal defences with particular reference to battered defendants [Report: NZLC r73]. Retrieved January 1, 2023, from https://www.lawcom.govt.nz/

[bibr37-10778012231192571] Lentz S. A. (1999). Revisiting the rule of thumb: An overview of the history of wife abuse. Women & Criminal Justice*,* 10(2), 9–27. 10.1300/J012v10n02_02

[bibr38-10778012231192571] Lion J. R. (1977). Clinical aspects of wife-battering. In Roy M. (Ed.), Battered women: A psychosociological study of domestic violence (pp. 126–136). Van Nostrand Reinhold Company.

[bibr39-10778012231192571] Loseke D. R. Gelles R.J. Cavanaugh M.M. (2005). Introduction: Understanding controversies on family violence. In Loseke D. R. Gelles R. J. Cavanaugh M. M. (Eds.), Current controversies on family violence (2nd ed., pp. ix–xix)*.* Sage Publications.

[bibr40-10778012231192571] Lovell T. (2000). Thinking feminism with and against Bourdieu. Feminist Theory, 1(1), 11–32. 10.1177/14647000022229047

[bibr41-10778012231192571] Marcus I. (1994). Reframing ‘domestic violence’: Terrorism in the home. In Fineman M. Mykitiuk R. (Eds.), The public nature of private violence: The discovery of domestic abuse (pp. 11–35). Routledge.

[bibr42-10778012231192571] McLeod J. (2005). Feminists re-reading Bourdieu: Old debates and new questions about gender habitus and gender change. Theory and Research in Education*,* 3(1), 11–30. 10.1177/1477878505049832

[bibr43-10778012231192571] McNay L. (1999). Gender, habitus and the field: Pierre Bourdieu and the limits of reflexivity. Theory, Culture & Society*,* 16(1), 95–117. 10.1177/026327699016001007

[bibr44-10778012231192571] Merriam-Webster. (n.d). Harm. In Merriam-Webster.com dictionary*.* Retrieved January 1, 2023, from https://www.merriam-webster.com/dictionary/harm

[bibr45-10778012231192571] Messner M. A. (2000). White guy habitus in the classroom: Challenging the reproduction of privilege. Men and Masculinities, 2(4), 457–469. 10.1177/1097184X00002004005

[bibr46-10778012231192571] Minow M. (1990). Words and the door to the land of change: Law, language, and family violence. Vanderbilt Law Review, 43(6), 1665–1699. https://scholarship.law.vanderbilt.edu/vlr/vol43/iss6/2

[bibr47-10778012231192571] Moore S. T. (2002). “Justifiable provocation”: Violence against women in Essex County, New York, 1799-1860. Journal of Social History*,* 35(4), 889–918. https://www.jstor.org/stable/3790615

[bibr48-10778012231192571] O'Neill D. (1998). A post-structuralist review of the theoretical literature surrounding wife abuse. Violence Against Women, 4(4), 457–490. 10.1177/1077801298004004005

[bibr49-10778012231192571] Ortiz-Barreda G. Vives-Cases C. (2013). Legislation on violence against women: Overview of key components. Revista panamericana de salud publica, 33(1), 61–72. 10.1590/s1020-49892013000100009 23440159

[bibr50-10778012231192571] Oxford English Dictionary. (n.d). Violence. In *oed.com dictionary* *.* Retrieved January 1, 2023, https://www.oed.com/oed2/00277885

[bibr51-10778012231192571] Pagelow M. D. (1981). Woman-battering. Sage Publications.

[bibr52-10778012231192571] Peterson del Mar D. (1992). Wife beating: An American tradition. Journal of Interdisciplinary History, 23(1), 97–118. 10.2307/205484

[bibr53-10778012231192571] Peterson del Mar D. (1996). What trouble I have seen: A history of violence against wives. Harvard University Press.

[bibr54-10778012231192571] Pleck E. (1983). Feminist responses to “crimes against women, 1868–1896. Signs: Journal of Women in Culture and Society, 8(3), 451–470. 10.1086/493985

[bibr55-10778012231192571] Pleck E. H. (2004). Domestic tyranny: The making of American social policy against family violence from colonial times to the present. University of Illinois Press.

[bibr56-10778012231192571] Ruether R. R. (1984). Church and family III: Religion and the making of the Victorian family. New Blackfriars, 65(765), 110–118. https://www.jstor.org/stable/43247487

[bibr57-10778012231192571] Schechter S. (1982). Women and male violence*.* South End Press.

[bibr58-10778012231192571] Schechter S. (1988). Building bridges between activists, professionals, and researchers. In Yllo K. Bograd M. (Eds.), Feminist perspectives on wife abuse (pp. 299–312)*.* Sage Publications.

[bibr59-10778012231192571] Sev’er A. (2002). Fleeing the house of horrors: Women who left abusive partners*.* University of Toronto Press.

[bibr60-10778012231192571] Sewell B. (1989). History of abuse: Societal, judicial, and legislative responses to the problem of wife beating. Suffolk University Law Review*,* XXIII*,* 983–1017.

[bibr61-10778012231192571] Shainess N. (1977). Psychological aspects of wife-battering. In Roy M. (Ed.), Battered women: A psychosociological study of domestic violence (pp. 111–119)*.* Van Nostrand Reinhold Company.

[bibr62-10778012231192571] Snell J. E. Rosenwald R. J. Robey A. (1964). The wifebeater’s wife: A study of family interaction. Archives of General Psychiatry, 11(2), 107–112. 10.1001/archpsyc.1964.01720260001001 14237606

[bibr63-10778012231192571] Stanton E. C. (1848). Declaration of sentiments. Retrieved on January 1, 2023, from https://www.womenshistory.org/sites/default/files/document/2019-08/Day%203_0.pdf

[bibr64-10778012231192571] Stark E. Flitcraft A. Frazier W. (1979). Medicine and patriarchal violence: The social construction of a “private” event. International Journal of Health Services, 9(3), 461–493. https://www.jstor.org/stable/45132090 468440 10.2190/KTLU-CCU7-BMNQ-V2KY

[bibr65-10778012231192571] Straus M. A. (1979). Measuring intrafamily conflict and violence: The conflict tactics (CT) scales. Journal of Marriage and the Family, 41(1), 75–88. 10.2307/351733

[bibr66-10778012231192571] Straus M. A. (2005). Women’s violence toward men is a serious social problem. In Loseke D. R. Gelles R. J. Cavanaugh M. M. (Eds.), Current controversies on family violence (pp. 55–77). Sage Publications.

[bibr67-10778012231192571] Tierney K. J. (1982). The battered women movement and the creation of the wife beating problem. Social Problems, 29(3), 207–220. 10.2307/800155

[bibr68-10778012231192571] Tomes N. (1978). A “torrent of abuse”: Crimes of violence between working-class men and women in London, 1840-1875. Journal of Social History*,* 11(3), 328–345. 10.1353/jsh/11.3.328

[bibr69-10778012231192571] Towns A. Adams P. (2000). If I really loved him enough, he would be okay. Violence Against Women*,* 6(6), 558–585. 10.1177/10778010022182038

[bibr70-10778012231192571] Webb J. T. Schirato T. Danaher G. (2002). Understanding Bourdieu. Sage.

[bibr71-10778012231192571] Wetherell M. Potter J. (1992). Mapping the language of racism: Discourse and the legitimation of exploitation. Harvester Wheatsheaf Publishers.

[bibr72-10778012231192571] Yllo K. (1988). Political and methodological debates in wife abuse research. In Yllo K. Bograd M. (Eds.), Feminist perspectives on wife abuse (pp. 28–50). Sage Publications.

[bibr73-10778012231192571] Yllo K. (2005). Through a feminist lens: Gender, diversity and violence – extending the feminist framework. In Loseke D. R. Gelles R. J. Cavanaugh M. M. (Eds.), Current controversies on family violence (pp. 19–34)*.* Sage Publications.

